# Design of Suspended Slot Racetrack Microring Refractive Index Sensor Based on Polymer Nanocomposite

**DOI:** 10.3390/polym15092113

**Published:** 2023-04-28

**Authors:** Xihan Wu, Jiajun Wang, Jiachen Han, Yuqi Xie, Xuyang Ge, Jianzhi Liao, Yunji Yi

**Affiliations:** 1 College of Integrated Circuits and Optoelectronic Chips, Shenzhen Technology University, Shenzhen 518118, China; 202100303055@stumail.sztu.edu.cn (X.W.);; 2 College of New Materials and New Energies, Shenzhen Technology University, Shenzhen 518118, China

**Keywords:** microring resonator, slot waveguide, polymer nanocomposites, integrated optics devices

## Abstract

Recently, polymer nanocomposites have attracted great interest due to their remarkable characteristics of high performance and enabling production of low-cost devices. This article explores the reflective index sensing application of the polymer nanocomposite IOC-133, which is a TiOx/polymer nanocomposite with a reflective index between 1.8 and 1.9. Considering the material properties of high reflective index, low absorption loss, and compatibility with nanoimprint lithography, a microring-based reflective index sensor with a suspended slot waveguide structure is proposed. We combined the sensing mechanism of slot waveguides with high reflective index polymer nanocomposites and designed the suspended structure to address the problem of decreasing sensitivity caused by residual layers. The sensing device was adopted as a microring resonator, which is conducive to large-scale integration. The finite-difference time-domain (FDTD) method was employed to analyze the effects of several key parameters. The results showed that the racetrack microring sensor we propose can achieve a high sensitivity of 436 nm/RIU (Refractive Index Units), about six times higher than the microring sensor with a ridge waveguide. The Q factor of the microring reaches 1.42 × 10^4^, and the detection limit is 1.38 × 10^−4^ RIU. The proposed suspended slot microring sensor has potential value in the field of nanoprinted photonic integrated circuits.

## 1. Introduction

In recent years, optical sensors have been widely used in biodetection [1], environmental detection [2], biochemical analysis [3], and other fields. Integrated optical waveguide sensors have attracted great interest owing to their small size, easy integration, and real-time dynamic response. They can also be applied in high-temperature environments and have the capability to be free of electromagnetic interference, which complements the vulnerability to electromagnetic interference and long sensing time of traditional electrical sensors [4,5]. Refractive index sensors are a significant type of liquid optical waveguide sensors that allow for highly sensitive detection and measurement of refractive index changes in the surrounding medium [6]. Optical liquid sensors convert the change in refractive index of an analyte into an optical signal via the interaction between light and the analyte, which achieves sensitive, real-time detection of the analyte [7].

There are a variety of materials utilized in integrated optical waveguide sensors, including silicon on insulator (SOI), III–V semiconductor materials, silicon dioxide (SiO_2_), and organic polymer materials. Most of the optical planar waveguide sensors have been realized based on the silicon-on-insulator (SOI) material platform owing to its high refractive index contrast and small size, which realize better array sensing functions [8,9]. Regarding the organic material, which is also an essential material in the field of optical waveguide sensors, polymers have attractive properties of flexibility, biocompatibility, stretchability, and cost-effective production compared to inorganic materials [10]. Based on the thermal curing property and photosensitivity of polymer materials, low-cost and simple fabrication processes such as direct photolithography [11], laser direct writing [12], and nanoimprinting [13] have been investigated. However, due to the inherent limitations of polymer materials, it is often necessary to change them using composite techniques to surpass these constraints and create innovative materials with enhanced performance [14]. Polymer nanocomposite is composed of a matrix material and a polymer as one of the constituents, along with a dispersed material such as single or multiple nanoparticles within the polymer matrix [15]. Different materials are combined chemically or physically to yield a composite material, offering an anticipated set of physio-chemical properties to accomplish the precise prerequisite for a certain application. Polymer nanocomposites hold great promise for improving the performance and functionality of various photonic and optoelectronic devices due to their high processability and exceptional functionalities.

The performance of optical waveguide sensors is intimately related to the material properties as well as the waveguide structures. Among varieties of influencing factors, the refractive index of the material is critical in affecting the sensing performance of optical waveguide sensors. Although polymer materials offer advantages in terms of material and processing costs, the sensing performance of the devices is limited by the poor optical field confinement due to its low refractive index, which is typically between 1.4 and 1.6 [16]. By increasing the refractive index of the polymer material, the mode size and bending loss of the guided light can be reduced, leading to a more compact device structure. Therefore, considerable effort has been invested in the development of high-refractive-index polymers and polymer composites [17]. IOC-133 (TiOx and polysiloxane), which is a polymer-TiOx nanocomposite with a reflective index between 1.8 and 1.9, can potentially exhibit superb performance in the sensing field due to its unique properties of relatively high refractive index, low nanofabrication process cost, and compatibility with high-resolution applications. Additionally, IOC-133 is able to operate at 850 nm wavelength, where aqueous solutions have low absorption loss, thus reducing absorption loss [18]. However, ridge waveguides and rectangular waveguides based on high-refractive-index polymers will lead to a weak evanescent field and thus decrease sensitivity to changes in the refractive index of the analyte. Slot waveguide sensors represent a superior choice, owing to their exceptional ability to guide and confine light within a nanometric index material region. Consequently, the combination of the sensing mechanism of slot waveguides with the properties of high-RI polymer nanocomposites is significant for optical waveguide sensing. Additionally, the compatibility of polymer nanocomposites with nanoimprint lithography offers a promising route towards the development of nanoscale devices in large-scale integration [19]. Meanwhile, it is important to stress that the residual slab layer that remains on the slab in the imprinting process can have adverse effects on the quality and accuracy of the structures as well as require increased processing costs for subsequent processing steps [20].

Beyond material properties and waveguides structure, the adoption of the device constitutes a crucial factor that influences the performance of a sensor. Up to now, varieties of polymer-based optical sensors have been developed, including Mach–Zehnder interferometer sensors [21], Young interferometer sensors [22], grating sensors [23], and microring resonator (MRR) sensors [24]. Among them, microring resonators do not require optical feedback provided by cavity surfaces or gratings for resonance, thus facilitating monolithic integration with other optoelectronic components [25]. In addition, microring resonators possess the characteristics of low cost, compact structure, high integration, low insertion loss, and low crosstalk [26]. In recent years, various structures of MRR sensors have been developed to improve their sensitivity. These structures include photonic crystal MRR [27], grating-type MRR [28], and slot microring resonator (SMRR) [29,30]. Besides their applications in such fields as optical signal processing [31], filtering [32], modulation [33], switching [34], and lasers [35], microrings also have great significance in the field of sensing [36]. Microring resonators have attracted a lot of attention from international researchers and have become a hot topic in sensing research. Ritu Raj Singh et al. proposed two ring resonators based on ridge and slot waveguides, respectively [37]. The results showed that the average sensitivity of slot ring resonators is observed to be 360 nm/RIU, which is six times higher than that of ridge ring resonators (60 nm/RIU). The ring resonator based on slot waveguides is proven to have a significant impact on the sensing field due to the strong optical field confinement effect, which exhibits more sensitive and accurate sensing performance compared to the ring resonator with ridge waveguide. In 2020, Ma et al. proposed a low-cost and highly sensitive liquid refractive index sensor based on polymer horizontal slot waveguides. The sensor demonstrated a sensitivity of 177 nm/RIU and a detection limit (DL) of 1.69 × 10^−4^ RIU [38].

In order to combine the sensing mechanism of slot waveguides with high-RI polymer nanocomposites and address the problem of decreasing sensitivity caused by residual layers, we designed a suspended slot waveguide microring reflective index sensor based on a polymer nanocomposite. The suspended structure was designed to enable the optical field of the slab to leak to the underlying analyte. A racetrack microring structure was adopted as the sensing device to ensure device compactness and simultaneously facilitate the potential for array integration. The average sensitivity of slot ring resonators proposed in this paper was observed to be seven times higher than that of typical microring resonators based on strip waveguides (60 nm/RIU) [39].

## 2. Structure Design

**Figure 1:**
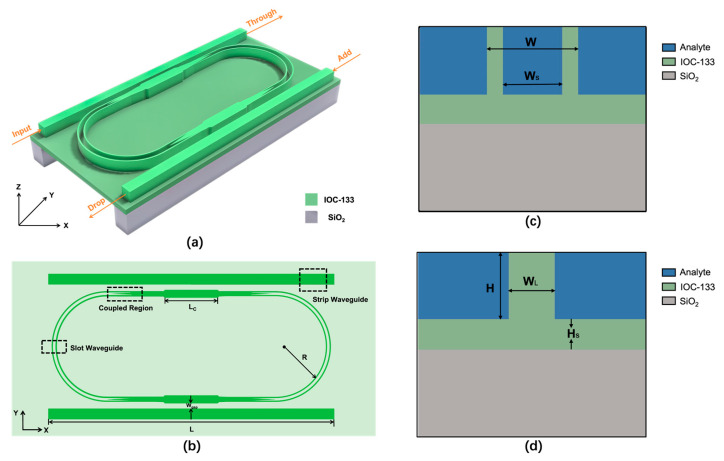
(**a**) Schematic diagram of the slot waveguide microring-based sensor. (**b**) The top view of the slot waveguide microring-based sensor. (**c**) Cross-sectional view of the slot waveguide. (**d**) Cross-sectional view of the strip waveguide.

The add-drop racetrack microring resonator where two parallel strip waveguides are coupled to a slot racetrack microring is depicted in Figure 1a. The polymer-based nanocomposite IOC-133, which has a refractive index of 1.87 at 850 nm wavelength, was adopted as the core material. The core layer was placed on top of the residual layer, which was generated during the nanoimprint process, and this configuration increased the contact area between the waveguides and the analyte. Liquid refractive index sensing was implemented in this paper, so the sensor device was immersed in aqueous solutions. Therefore, when optimizing the geometrical parameters of waveguides, pure water with a refractive index of 1.33 was selected as the cladding. Several essential geometrical parameters of the microring are noted on the top view of the microring in Figure 1b. The microring radius R represents the distance between the center of the ring and the center of the slot waveguide and was set to 50 µm. The gap width between the bus waveguides and the ring $W_{gap}$ was set to 0.1 µm, resulting in lower transmission loss. The lengths of the upper and lower bus waveguides L and the length of racetrack coupling region $L_{C}$ were selected as 270 µm and 40 µm, respectively. A tapered mode converter was utilized to realize the mode conversion between the straight waveguide and the slot waveguide [40]. In addition, Figure 1c,d illustrates the cross-sectional views of the slot waveguide and strip waveguide, respectively. In order to maintain single-mode transmission and enhance the coupling effect with the microring resonator, the strip waveguide width $W_{L}$ was chosen as 0.4 µm. The height of the core layer H was fixed at 1 µm owing to the material properties in the spin-coating process. The thickness of the residual $H_{S}$ was set to 100 nm due to the precision of the nanoimprint. To achieve strong restriction of the optical field, the total waveguide width W and the slot width $W_{S}$ were set to 210 nm and 150 nm, respectively.

## 3. Stimulation and Analysis

### 3.1. Structure Optimization

The finite difference time-domain (FDTD) method was used to optimize the sensing performance of these microring-based liquid sensors with suspended slot waveguide [41]. The sensor proposed was designed using Lumerical’s FDTD solutions, the fundamental transverse magnetic (TM) mode was injected into the input port of the bus waveguide from the mode source. To achieve higher Q-factor and sensitivity, the selection of polarization state, optimization of waveguide parameters, and analysis of waveguide modes with respect to the waveguide structure were discussed. In terms of the device structure, the transmission loss, coupling efficiency, and mode conversion were analyzed.

The influence of the working polarization state of the optical wave on the sensing sensitivity was analyzed. The refractive index sensitivity of the microring sensor is defined as the shift in resonant wavelength as a function of the change in refractive index of the cladding:

(1) $$S=\frac{\partial \lambda_{m}}{\partial n_{en}}=\frac{\partial \lambda_{m}}{\partial n_{eff}}\frac{\partial n_{eff}}{\partial n_{en}}=\frac{\lambda_{m}}{n_{eff}}\frac{{\partial n}_{eff}}{\partial n_{en}}$$

where $\lambda_{m}$ is the resonant wavelength, $n_{en}$ is the cladding refractive index, and $n_{eff}$ is the effective refractive index of the waveguide.

**Figure 2:**
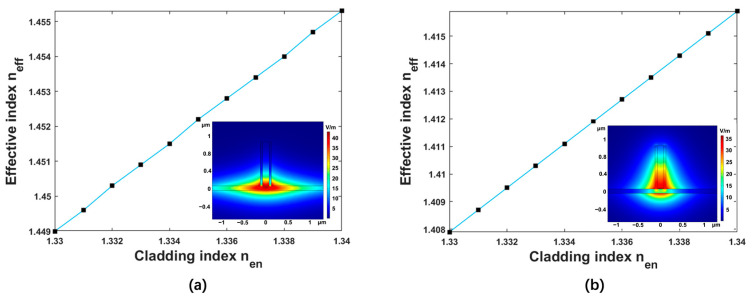
(**a**) The $n_{eff}$ as functions of $n_{en}$ for TE mode. (**b**) The $n_{eff}$ as functions of $n_{en}$ for TM mode. Insets: the distribution of the optical fields for TE mode and TM mode, respectively.

High sensitivity indicates that the waveguide detects the changing refractive index of the analyte more precisely. The polarization state of the optical wave directly affects the sensing sensitivity. Thus, to obtain high sensitivity, the appropriate selection of polarization state for optical wave operation is of great significance. The waveguide height, the slot width, and the total waveguide width were fixed as H = 1 μm, $W_{S}$ = 150 nm and W = 250 nm, respectively. The variations of the effective refractive index of the waveguide with the refractive index of the analyte in the transverse electric mode (TE) and transverse magnetic mode (TM) are shown in Figure 2. The sensitivities of the waveguide for the TE and TM polarizations were 343.02 nm/RIU and 435.58 nm/RIU, respectively. The optical power ratio Ƞ represents the cladding optical field energy divided by the total energy, which can reflect the intensity of the interaction between the optical field and analyte. The values of Ƞ for TE and TM polarization were 80.9% and 87.5%, respectively. Therefore, both the sensitivity and the Ƞ of the slot waveguide for the TM polarization were higher than that for the TE polarization. The distribution of the optical field showed that there was an optical field leakage in the slot waveguide for TE polarization, and the slot waveguide for TM polarization exhibited stronger confinement on the optical field. Therefore, the TM polarization was selected as the working polarization of the optical wave, and the following analyses were performed at the TM polarization. Additionally, the sensitivity and Ƞ of the suspended slot waveguide and slot waveguide on substrate for TM polarization were analyzed, respectively. The sensitivity of the slot waveguide on substrate for TM polarization was 320 nm/RIU, and the Ƞ was 57.6%. The sensitivity of the suspended slot waveguide was 1.36 times higher than that of slot waveguide on substrate, and the optical power ratio was 1.52 times higher than the non-suspended structure. The adoption of a suspended structure had a positive impact on sensitivity enhancement.

**Figure 3:**
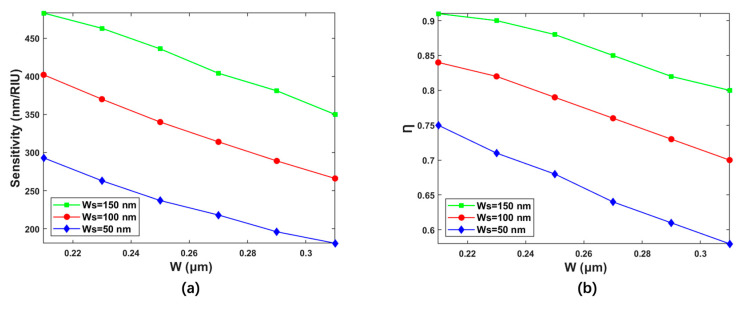
(**a**) Relationships between the sensing sensitivity and the wavelength at different slot widths. (**b**) Relationships between the Ƞ and the wavelength at different slot widths.

The structural parameters that have great influence on the waveguide performance include the slot width and the total waveguide width. The effects of the variations of these two structural parameters on the waveguide performance were analyzed, respectively. The waveguide height was selected as H = 1 μm due to the material properties in the spin-coating process. Figure 3 shows the variation of slot sensing sensitivity and Ƞ with W for different $W_{S}$ of 50 nm, 100 nm, and 150 nm. As shown in Figure 3a, with W changing from 210 nm to 310 nm, the corresponding values of sensitivity were 293 nm/RIU–181 nm/RIU, 402 nm/RIU–266 nm/RIU, and 483 nm/RIU–310 nm when $W_{S}$ was selected to be 50 nm, 100 nm, and 150 nm, respectively. Figure 3b exhibits the variation of the Ƞ with the W for different $W_{S}$. The values of Ƞ vary from 74.81% to 58.43%, 84.15% to 70.05%, and 90.98% to 79.72% for $W_{S}$ of 50 nm, 100 nm, and 150 nm, respectively. It can be concluded that the sensitivity and Ƞ of the waveguide increase with the increase in $W_{S}$, and decrease with the increase in W. Therefore, the slot width $W_{S}$ of 150 nm with the maximum sensitivity and Ƞ was adopted for further study.

To meet the single-mode condition of the slot waveguide and improve the sensing performance, the mode number, optical field distribution, and performance parameters of the waveguide with different total waveguide widths were simulated. The waveguide height and slot width were adopted to be H = 1 μm and $W_{S}$ = 150 nm.

**Figure 4:**
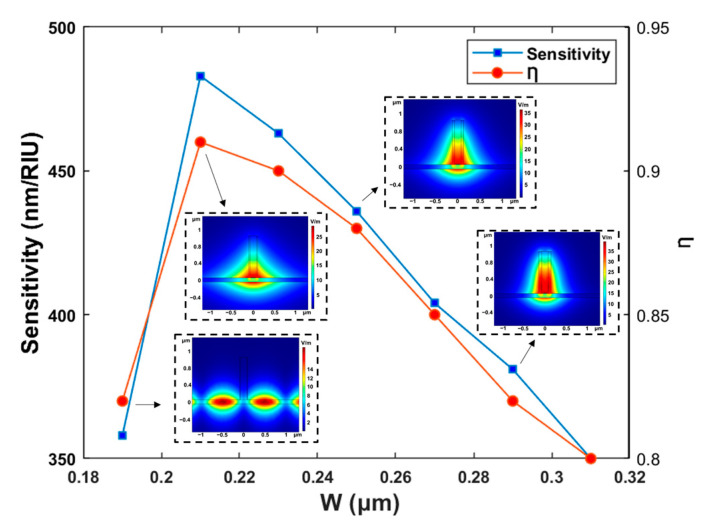
The sensing sensitivity as functions of wavelength. Insets: the light field distribution when W = 190 nm, W = 210 nm, W = 250 nm, W = 290 nm, respectively.

**Figure 5:**
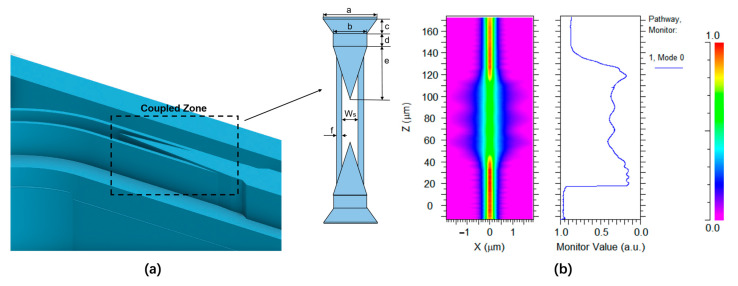
(**a**) Schematic of the mode converter. (**b**) The stimulated optical field of the mode converter.

Figure 4 illustrates the influence of the total waveguide width W on sensitivity and Ƞ, and the optical field distribution corresponding to W = 190 nm, W = 210 nm, W = 250 nm, and W = 290 nm. When W was selected as 190 nm, the waveguide was unable to meet the single-mode condition and form slot waveguides. When W was adopted to be 210 nm, the optical field leaked from the slot waveguide, which led to a poor optical power ratio. Stable single-mode operation was produced when W was selected as 250 nm, and there was no significant influence on the optical field distribution when increasing W to 290 nm. In addition, the data in Figure 5 show that the sensitivity and Ƞ of the waveguide decreased with the increase in the total width W of the waveguide. The W = 250 nm was adopted to form a stable slot waveguide mode with high sensitivity and Ƞ.

The slot waveguide was adopted for the proposed microring-based sensor to improve the sensitivity of the sensor. However, the coupling loss and transmission loss of the slot waveguide were higher than those of the straight waveguide due to its non-Gaussianity. Therefore, an efficient mode converter was adopted to achieve the mode conversion from the strip waveguide into the slot waveguide. The diagram of the tapered mode converter is shown in Figure 5a. To reduce the mode mismatch and improve the coupling efficiency, taking the structural parameters, the posterior end of taper width a= 0.4 μm, the fore part of taper width b = 0.32 μm, the length of taper c = 8 μm, the length of the transition waveguide d = 13 μm, the length of the slot-inserted taper e = 42 μm, the width of the slot waveguide $W_{s}$ = 0.15 μm, and the waveguide width on both sides of the slot f = 0.05 μm. The energy loss was 0.38 dB, and the optical field distribution is shown in Figure 5b.

**Figure 6:**
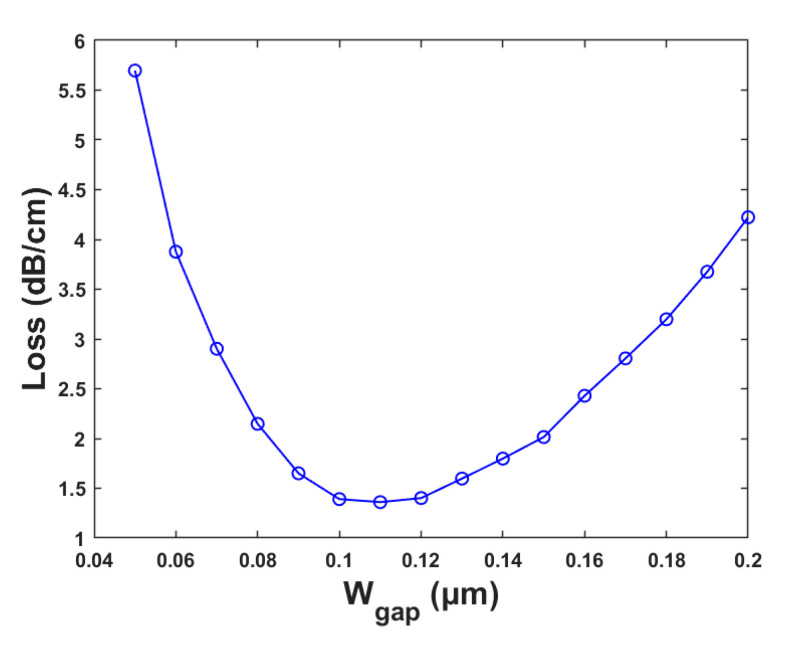
Relationships between Loss and the $W_{gap}$.

To improve the coupling effect and reduce the transmission loss, the width of the strip waveguide $W_{L}$ was set to 0.4 μm, and the coupling length $L_{C}$ was selected as 50 μm. The central coupling gap between the channels and the microring is a key factor affecting the output spectrum shape of the device. Excessive $W_{gap}$ will result in low coupling efficiency and high loss when the majority of the light is transmitted through the straight waveguide rather than the microring. The coupling matrix is only satisfied in the section near the microring. However, it is challenging to realize the coupled region with a too small coupling gap by the existing process, which requires a high preparation cost as well. The coupling gap was optimized to decrease the transmission loss. Figure 6 shows the curve of the loss versus the coupling gap. The coupling spacing $W_{gap}$ was selected to be 0.11 μm with the minimum loss of 1.36 dB/cm.

### 3.2. Performance Analysis

The microring-based sensing was conducted by tracking the resonant wavelength shift. To achieve high sensing sensitivity, wavelength shift must be detected quickly and precisely, which necessitates reasonable amplitude coupling coefficients. The transmission characteristics of the drop port is recorded as

(2) $$T_{D}=\frac{{\left|k_{1}\right|}^{2}{\left|k_{2}\right|}^{2}\alpha }{1-2\alpha \left|t_{1}\right|\left|t_{2}\right|\left|\cos\theta +\alpha^{2}\right|{\left|t_{1}\right|}^{2}{\left|t_{2}\right|}^{2}}$$

$k_{1}$ and $k_{2}$ represent, respectively, the field coupling coefficients in different regions. $t_{1}$ and $t_{2}$ are the field transmission coefficients in corresponding regions. α is transmission loss coefficient. θ is the phase of optical wave transmission in the microring. The light path can be reversed because of the symmetry of the entire structure in the y-direction. The relationship between $k_{1}$, $t_{1}$, $k_{2}$ and $t_{2}$ can be written as

(3) $${\left|k_{1}\right|}^{2}+{\left|t_{1}\right|}^{2}=1$$

(4) $${\left|k_{2}\right|}^{2}+{\left|t_{2}\right|}^{2}=1$$

**Figure 7:**
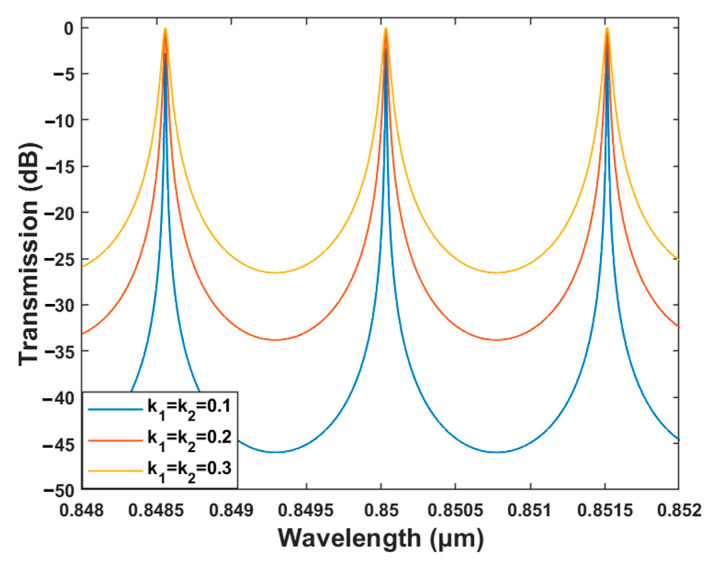
Transmission characteristics at the drop port of microring resonator with different amplitude coupling ratios.

Under the phase resonance condition, the resonant light intensity in the microring resonator is maximized when the coupling coefficients $k_{1}$ and $k_{2}$ are symmetrically equal. The resonant wavelength output in the drop port reaches its maximum, while the resonant wavelength in the through port is completely absorbed by the microring resonator. To eliminate this extra loss and decrease the optical intensity of the non-resonant signal, the same amplitude coupling ratio between the microring and the channels is selected. When shifting of the resonant peak is caused by changes in the material, technique, and temperature of the device, the sensitivity of the device will be affected. Figure 7 illustrates the influence of different amplitude coupling ratios on the output spectrum. When the amplitude coupling ratio increases, the output optical power at a non-resonance wavelength will be greater. The excessively wide resonant peak caused by the large amplitude coupling ratio will reduce the sensitivity of the sensor in detecting the resonant wavelength shift. By decreasing the amplitude coupling ratio, cross-talk can be reduced or prevented and a narrow resonant peak can be obtained.

The quality factor is a significant property of resonator-based sensors, which reflects the sensitivity of the device. The Q factor is defined as the ratio of resonance wavelength ($\lambda_{res}$) to full width at half the maximum of the peak of the resonance (${∆\lambda }_{FWHM}$), that is

(5) $$Q=\frac{\lambda_{m}}{{△\lambda }_{FWHM}}$$

When the amplitude coupling ratio between the microring and the channels are selected to be 0.1, 0.2, and 0.3, the corresponding Q factors are 1.42 × 10^4^, 6.81 × 10^3^, and 3.96 × 10^3^, respectively. Moreover, the sensing detection capability of the sensor is affected by both the demodulation system and environment. The detection limit (DL) is a significant factor in determining whether the sensor can be widely used in practical sensing applications. For liquid sensing, DL is defined as the smallest physical quantity of the analyte that can be accurately quantified by a refractive-index-based sensor.

(6) $$DL=\frac{\lambda_{res}}{QS}$$

A low DL indicates high sensing and detection specifications for the sensor. The corresponding DL are 1.38 × 10^−4^, 2.87 × 10^−4^, and 4.93 × 10^−4^ when the same amplitude coupling ratios are equal to 0.1, 0.2, and 0.3, respectively. Therefore, considering the Q factor and the DL of the sensor, the amplitude coupling ratio was selected to be 0.1.

**Figure 8:**
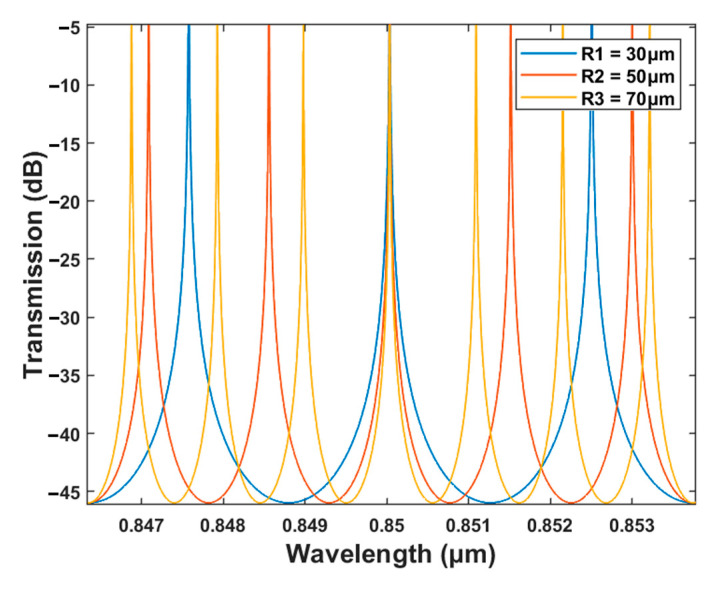
Transmission characteristics at the drop port of the microring resonator under different radiuses.

With the great advantages of the microring resonator, it is crucial to investigate the influence of the microring radius on the output spectrum. The transmission characteristics at the drop port of microring resonators with R = 30 μm, R = 50 μm, and R = 70 μm are shown in Figure 8, respectively. As the radius of the microring resonator increases, the effective coupling range expands due to the more uniform modal field distribution within the ring waveguide. Moreover, the increasing radius enhances the coupling effect between the ring and strip waveguides and enables more light to be effectively coupled into the ring cavity. However, an oversized microring resonator necessitates a larger space to accommodate the ring structure, leading to a larger device footprint that results in increased integration difficulty. Therefore, in this paper, a microring radius of 50 μm was adopted.

**Figure 9:**
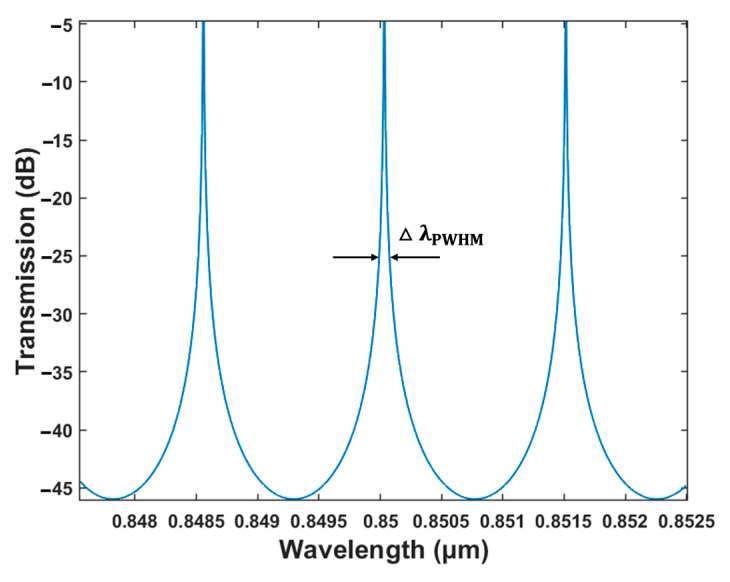
Simulated transmission spectra at the drop port of the microring.

Figure 9 depicts the transmission spectrum of the drop port of the microring-based sensors with slot waveguides. A microring with a 3 dB bandwidth of 0.34 nm, a Q factor of 1.42 × 10^4^, and a DL of 1.38 × 10^−4^ RIU was ultimately obtained.

The results of the suspended slot microring sensor we designed were compared with those of the reported microring resonator, as shown in Table 1. In recent years, the sensitivity of sensors based on microring resonators has been typically around 10^2^. In the sensing field, it has been found that the slotted microring resonator (SMRR) outperforms the microring resonator MRR with strip waveguides. In comparison to the SOI MRR with strip waveguides, the sensitivity of the SOI SMRR is significantly higher due to the increased confinement on the optical field. The polymer-based SMRR also exhibits superior performance compared to the polymer-based MRR with strip waveguides. The suspended microring based on slot waveguides in this study, which adopted polymer nanocomposites as the core material, outperformed existing polymer-based SMRR in terms of sensitivity while having comparable sensitivity with SOI SMRR.

**Table 1:** Comparison of different microring resonator sensors.

Reference	Structure	Material	Ws (nm)	Q-Factor	DL (RIU)	S (nm/RIU)
[41]	SOI MRR	Si	\	20,000	10 ng/mL	70
[32]	SOI SMRR	Si	140	1113	0.129%	403
[27]	SOI SMRR	Si	200	3000	8.8 × 10^−6^	240
[28]	MRR	Polymer	\	15,000	\	115 ± 8
[40]	SMRR	Polymer	200	\	1.69× 10^−4^	177
Our work	SMRR	Composites	150	14,200	1.38 × 10^−4^	436

## 4. Conclusions

In this paper, we propose a microring refractive index sensor with a suspended slot waveguide based on polymer nanocomposites. We combined the sensing mechanism of slot waveguides with high-RI polymer nanocomposites and designed the suspended structure to address the problem of decreasing sensitivity caused by residual layers. The suspended structure can substitute the dry etching process with the wet etching process for the substrate, reducing the potential process cost and device roughness. A microring was adopted as the sensing device to take advantage of the compatibility of polymer composite materials with nanoimprint lithography, thus offering a promising route towards large-scale integration. The polarization state and the structural parameters of the slot waveguide were optimized by analyzing the sensing performance and single-mode conditions. Tapered mode converters were adopted in the waveguide mode conversion to reduce the translation losses. Moreover, the coupling spacing was adjusted to enhance the coupling effect between the bus waveguides and the microring, and the transmission loss was reduced to 1.36 dB/cm. The bending radius and coupling efficiency of the microring sensor were selected by investigating the transmission characteristics. The results showed that the microring-based sensor with a radius of 50 μm can achieve a high sensitivity of 436 nm/RIU, which is about 1.36 times higher than the slot microring on substrate and six times higher than the microring sensor with strip waveguides. The quality factor of the sensor reached 1.42 × 10^4^, and the detection limit was 1.38 × 10^−4^ RIU. The proposed suspended microring sensor is expected to apply in the field of low-cost liquid sensors using nanoimprinting techniques.

## Data Availability

The data that support the findings of this study are available from the corresponding author upon reasonable request.

## References

[1] Konopsky V.N., Alieva E.V. (2019). Imaging biosensor based on planar optical waveguide. Opt. Laser Technol..

[2] Sader E., Sayyed-Ahmad A. (2013). Design of an optical water pollution sensor using a single-layer guided-mode resonance filter. Photonics Sens..

[3] Cunningham B., Li P., Bo L., Pepper J. Colorimetric Resonant Reflection as a Direct Biochemical Assay Technique. Proceedings of the Paper Presented at the Fifteenth IEEE International Conference on Micro Electro Mechanical Systems.

[4] Snelders D.J.M., Boersma A. (2014). Development of thermostable FBG optical sensor for oil and gas applications. Int. J. Smart Sens. Intell. Syst..

[5] Esequiel M., Luís P., Andreas T., Nélia A., José M., Carlos M., Kyriacos K., Paulo A., Humberto V., Paulo A. (2018). Optical Sensors for Bond-Slip Characterization and Monitoring of Rc Structures. Sens. Actuators A Phys..

[6] Ma X.X., Chen K.X., Wu J.Y. (2020). Cost-Effective Mach-Zehnder Interferometer Liquid Refractive Index Sensor Based on Conventional Polymer Strip Waveguide. IEEE Photonics J..

[7] Tsigaridas G.N. (2017). A study on refractive index sensors based on optical micro-ring resonators. Photonics Sens..

[8] Elsayed M.Y., Sherif S.M., Aljaber A.S., Swillam M.A. (2020). Integrated Lab-on-a-Chip Optical Biosensor Using Ultrathin Silicon Waveguide SOI MMI Device. Sensors.

[9] Donzella V., Sherwali A., Flueckiger J., Grist S.M., Fard S.T., Chrostowski L. (2015). Design and fabrication of SOI micro-ring resonators based on sub-wavelength grating waveguides. Opt. Express.

[10] Ma H., Jen A.K., Dalton L.R. (2010). Polymer-Based Optical Waveguides: Materials, Processing, and Devices. Adv. Mater..

[11] Fiedorowicz H., Bartnik A., Jarocki R., Kostecki J., Rakowski R., Szczurek M. Micromachining of organic polymers by direct photo-etching using a compact laser plasma soft x-ray source. Proceedings of the Paper presented at the Conference on Nanoengineering: Fabrication, Properties, Optics, and Devices II.

[12] Liang S., Yang Y., Lv C., Liu Y., Xia H. (2022). Integratable photodetectors based on photopolymerized conductive polymer via femtosecond laser direct writing. Opt. Lett..

[13] Wang X., Bhadauriya S., Zhang R., Pitliya P., Raghavan D., Zhang J., Bockstaller M.R., Douglas J.F., Karim A. (2019). Nanoimprint Directed Assembly of Associating Polymer-Grafted Nanoparticles for Polymer Thin Films with Enhanced Stability. ACS Appl. Polym. Mater..

[14] Shukla P., Saxena P. (2021). Polymer Nanocomposites in Sensor Applications: A Review on Present Trends and Future Scope. Chin. J. Polym. Sci..

[15] Kanoun O., Bouhamed A., Ramalingame R., Bautista-Quijano J.R., Rajendran D., Al-Hamry A. (2021). Review on Conductive Polymer/CNTs Nanocomposites Based Flexible and Stretchable Strain and Pressure Sensors. Sensors.

[16] Yasir M., Sai T., Sicher A., Scheffold F., Steiner U., Wilts B.D., Dufresne E.R. (2021). Enhancing the Refractive Index of Polymers with a Plant-Based Pigment. Small.

[17] Song D.P., Li C., Li W., Watkins J.J. (2016). Block Copolymer Nanocomposites with High Refractive Index Contrast for One-Step Photonics. ACS Nano.

[18] Niu D., Zhang D., Yang K., Lian T., Sun S., Li B., Wang X. (2020). 850-nm polymeric waveguide thermo-optic switch with low power-consumption. Opt. Laser Technol..

[19] Morarescu R., Pal P.K., Beneitez N.T., Missinne J., Steenberge G.V., Bienstman P., Morthier G. (2016). Fabrication and Characterization of High-Optical-Quality-Factor Hybrid Polymer Microring Resonators Operating at Very Near Infrared Wavelengths. IEEE Photonics J..

[20] Maxwell A., Huang S.-W., Ling T., Kim J.-S., Ashkenazi S., Guo L.J. (2008). Polymer Microring Resonators for High-Frequency Ultrasound Detection and Imaging. IEEE J. Sel. Top. Quantum Electron..

[21] Zhang J., Li Y., Jiang C., Zhao Z. (2021). Optical Waveguide Electric Field Sensor Based on Dual Parallel Mach-Zehnder Interferometer. IEEE Sens. J..

[22] Hiltunen M., Hiltunen J., Stenberg P., Aikio S., Kurki L., Vahimaa P., Karioja P. (2014). Polymeric slot waveguide interferometer for sensor applications. Opt. Express.

[23] Mattelin M.A., Missinne J., Coensel B.D., Steenberge G.V. (2020). Imprinted Polymer-Based Guided Mode Resonance Grating Strain Sensors. Sensors.

[24] Liang Y., Liu Q., Wu Z., Morthier G., Zhao M. (2019). Cascaded-Microrings Biosensors Fabricated on a Polymer Platform. Sensors.

[25] Girault P., Lorrain N., Poffo L., Guendouz M., Lemaitre J., Carré C., Gadonna M., Bosc D., Vignaud G. (2015). Integrated polymer micro-ring resonators for optical sensing applications. J. Appl. Phys..

[26] Gylfason K.B., Carlborg C.F., Kaźmierczak A., Dortu F., Sohlström H., Vivien L., Barrios C.A., van der Wijngaart W., Stemme G. (2010). On-chip temperature compensation in an integrated slot-waveguide ring resonator refractive index sensor array. Opt. Express.

[27] Xu Y., Hu S., Kong M. (2020). Air-Mode Photonic Crystal Micro-Ring Resonator With Enhanced Quality Factor for Refractive Index Sensing. IEEE Photonics J..

[28] Tu Z., Gao D., Zhang M., Zhang D. (2017). High-sensitivity complex refractive index sensing based on Fano resonance in the subwavelength grating waveguide micro-ring resonator. Opt. Express.

[29] Ahmadian D., Ghoabdi C., Nourinia J. (2015). Tunable Plasmonic Sensor With Metal–Liquid Crystal–Metal Structure. IEEE Photonics J..

[30] Shi B., Chen X., Cai Y., Zhang S., Wang T., Wang Y. (2022). Compact Slot Microring Resonator for Sensitive and Label-Free Optical Sensing. Sensors.

[31] Huang C., Jha A., de Lima T.F., Tait A.N., Shastri B.J., Prucnal P.R. (2021). On-Chip Programmable Nonlinear Optical Signal Processor and Its Applications. IEEE J. Sel. Top. Quantum Electron..

[32] Tamada A., Ota Y., Kuruma K., Watanabe K., Iwamoto S., Arakawa Y. (2019). Single Plasmon Generation in an InAs/GaAs Quantum Dot in a Transfer-Printed Plasmonic Microring Resonator. ACS Photonics.

[33] Bahadori M., Goddard L.L., Gong S. (2020). Fundamental electro-optic limitations of thin-film lithium niobate microring modulators. Opt. Express.

[34] Wang Y., Xi G., Zhang Y., Song M., Liu C., Wang G., Wang C., Ma N., Qin Z. (2020). Evaluation of a cross-grid microring resonator electro-optic switching array. Optik.

[35] Zhu Y., Zhao Y., Zhu L. (2017). Modal Discrimination in Parity-Time-Symmetric Single Microring Lasers. IEEE Photonics J..

[36] Wan L., Chandrahalim H., Chen C., Chen Q., Mei T. (2017). On-Chip, High-Sensitivity Temperature Sensors Based on Dye-Doped Solid-State Polymer Microring Lasers. Appl. Phys. Lett..

[37] Singh R.R., Kumari S., Gautam A., Priye V. (2018). Glucose Sensing Using Slot Waveguide-Based SOI Ring Resonator. IEEE J. Sel. Top. Quantum Electron..

[38] Ma X., Chen K., Wu J., Wang L. (2020). Low-Cost and Highly Sensitive Liquid Refractive Index Sensor Based on Polymer Horizontal Slot Waveguide. Photonic Sens..

[39] De Vos K., Bartolozzi I., Schacht E., Bienstman P., Baets R. (2007). Silicon-on-Insulator microring resonator for sensitive and label-free biosensing. Opt. Express.

[40] Han J., Wu X., Ge X., Xie Y., Song G., Liu L., Yi Y. (2022). Highly Sensitive Liquid M-Z Waveguide Sensor Based on Polymer Suspended Slot Waveguide Structure. Polymers.

[41] Nguyen D.T., Norwood R.A. (2013). Label-free, single-object sensing with a microring resonator: FDTD simulation. Opt. Express.

